# Assessing the Impact of Genratest on Women With Recurrent Implantation Failure: A Single-Center Study

**DOI:** 10.7759/cureus.52256

**Published:** 2024-01-14

**Authors:** Huy Phuong Tran, Loc Thai Ly, Vy Nguyen-Thao Do, Tuyet Thi-Diem Hoang, Thuy Thi-Thanh Tran, Hien Nguyen-Trong Le, Phuong Thi-Vy Nguyen, Ngoc Anh Nguyen, Trang Nguyen-Khanh Huynh

**Affiliations:** 1 Infertility Department, Hung Vuong Hospital, Ho Chi Minh, VNM; 2 Medical Genetics Department, Hung Vuong Hospital, Ho Chi Minh, VNM; 3 Obstetrics and Gynaecology Department, Pham Ngoc Thach University of Medicine, Ho Chi Minh, VNM

**Keywords:** genratest, recurrent implantation failure, embryo transfer, in vitro fertilization, ivf

## Abstract

Objective

Recurrent implantation failure (RIF) is a significant challenge in assisted reproduction. Genratest has emerged as a potential tool to identify the displaced window of implantation (WOI). This study aimed to evaluate the impact of this test on the pregnancy outcomes of RIF patients.

Methods

A retrospective analysis was conducted on 143 RIF patients who were categorized into two groups: the personalized embryo transfer (pET, n=69) group and standard embryo transfer (sET, n=74) group. The main measured outcomes were clinical pregnancy, ongoing pregnancy, miscarriage, and live birth rates.

Results

Genratest effectively diagnoses the displaced WOI in 90% of RIF patients. The pET group exhibited a higher rate of clinical pregnancy (n=36/69, 52.2%) compared to the sET group (n=35/74, 47.3%), but this difference was not statistically significant (p=0.679). Ongoing pregnancy rates were comparable between the pET (n=28/69, 40.6%) and the sET (n=30/74, 40.5%) groups (p=0.996). Live birth rates showed no statistically significant difference between the two groups (n=26/69, 37.7% versus n=22/74, 29.7%, p=0.407). Miscarriage rates were similar in both groups (n=9/69, 13% versus n=11/74, 14.9%, p=0.942).

Conclusions

pET based on the results of the Genratest did not show a significant improvement in pregnancy outcomes, including clinical pregnancy, ongoing pregnancy, live birth, or miscarriage rates. Further research is needed to identify the role of Genratest in RIF patients.

## Introduction

Recurrent implantation failure (RIF) is a severe problem for couples attempting to conceive with assisted reproductive procedures (ART). It is defined by the failure of embryos to implant and establish a pregnancy within the uterus. RIF is a frustrating experience for couples as it delays their path to motherhood and frequently involves emotional and financial challenges. This condition arises from combined factors, including maternal, embryonic, and uterine variables. For example, endometrial abnormalities, decreased endometrial receptivity, embryo-related factors, immunological factors, or hormonal factors are among the factors identified in a related study [1]. In some cases, RIF may occur without a clear explanation. RIF treatment focuses on addressing the fundamental causes found in each situation. These may include hormonal supplementation, endometrial preparation protocols, surgical interventions to correct uterine abnormalities, immune-modulating therapies, or genetic testing of embryos to select the healthiest embryos for transfer [1]. However, it is worth noting that there are varying opinions regarding RIF and the efficacy of certain interventions. Some argue that RIF may be seen as a condition that prompts patients to spend extra money on add-on treatments [2].

During the menstrual cycle, the uterine lining in the endometrium undergoes dynamic modifications to produce an optimum environment for embryo implantation. A competent embryo and a prepared endometrium are two critical factors in successful embryonic implantation. Understanding this complicated mechanism has recently become a focus of research [3]. The failure of the endometrium to attain a receptive state or window of implantation (WOI) at the time of embryo transfer is considered a critical cause of RIF. As a result, determining the endometrium's receptivity status has emerged as a viable technique for increasing the chances of successful implantation and subsequent pregnancy.

Genratest is a molecular diagnostic tool that provides detailed insights into the gene expression profile of the endometrium [4]. By analyzing the transcriptomic signature of 238 genes of endometrial tissue, the Genratest can accurately determine the precise period when the endometrium is most favorable for embryo implantation [5]. The ultimate goal is to improve reproductive outcomes and provide targeted interventions for couples undergoing ART. This personalized strategy helps overcome the limitations of relying solely on standardized protocols and increases the likelihood of successful implantation. Several studies have investigated the utility of Genratest in women experiencing RIF, aiming to determine its impact on pregnancy rates [6,7].

Despite the theoretical promise of Genratest in addressing RIF, its effectiveness in improving pregnancy outcomes remains a subject of debate [8,9]. Current meta-evidence also does not support the use of Genratest. For example, our meta-analysis published in 2021 concluded that Genratest shows no significant improvement in IVF outcomes [10]. Recently, another updated review also concurred that Genratest appears to possess limited guidance in embryo transfer [11]. As a result, the question remains whether misplaced endometrial receptivity can be a cause of RIF and whether embryo transfer guided by Genratest improves reproductive outcomes in a group of RIF patients. Moreover, Genratest is a specialized and relatively expensive procedure. Its availability may be limited to certain clinics or regions, and the cost may be an important consideration for some couples.

To contribute to this ongoing research and to gain more insight into the effectiveness of Genratest-guided personalized embryo transfer, we conducted a retrospective study involving a population of RIF patients. In this study, we aimed to evaluate the impact of personalized embryo transfer (pET) based on Genratest results on pregnancy outcomes of RIF patients, focusing on clinical pregnancy, ongoing pregnancy, miscarriage, and live birth rates.

## Materials and methods

Study design and population

This single-center retrospective cohort study was conducted at the Infertility Department of Hung Vuong Hospital, Ho Chi Minh City, Vietnam. Data was collected using medical records between January 2021 to December 2021. We included patients with an RIF history. It is worth noting that the RIF definition varied among centers. According to Hung Vuong Hospital, RIF was diagnosed when no clinical pregnancy occurred after at least two consecutive embryo transfers with good-quality embryos. The reproductive outcomes of the experimental group after pET were compared to reproductive outcomes in the control group after standardized embryo transfer (sET). We excluded the donor cycle and cycle with preimplantation genetic testing.

Study protocol

We used artificial hormone replacement to prepare the endometrium for both the mock and transfer cycles. Particularly, on days two to three of the cycle, patients began taking 2 mg of oral estradiol (Valiera 2 mg or Progynova 2 mg) twice a day. The dosage was increased every five days in 4 mg intervals, up to a maximum of 16 mg daily. For the next 14-16 days, transvaginal sonography was used to assess the pattern and thickness of the endometrium. Once the endometrial thickness reached 8-14 mm with a triple-line pattern, luteal support was initiated with vaginal administration of progesterone using Utrogestan 200 mg. The initial day of progesterone administration was labeled as P+0, and a biopsy was performed using a Pipelle catheter after five full days of progesterone administration (P+5).

After the biopsy, the endometrial tissue was transferred to a cryotube and kept at 4°C. The samples were then shipped at room temperature to GENTIS Company for Genratest analysis to identify the WOI. The Genratest result can be either receptive or non-receptive. A receptive endometrium indicates a good moment for embryo transfer. A non-receptive endometrium, on the other hand, implies a pre-receptive or post-receptive moment, which is not optimal for embryo transfer. The embryo is transferred in the following cycle when the Genratest confirms the precise WOI time. We used morphological criteria to determine the quality of the transferred embryos [12].

Pregnancy outcomes were documented. In this study, clinical pregnancy was defined by the presence of a viable fetal heart rate and crown-rump length (CRL) of ≥7 mm, as determined between seven and eight weeks of gestation. Ongoing pregnancy was categorized as pregnancies that persisted beyond 12 weeks of gestation. Live births refer to the total number of successfully delivered babies who reached a gestational age of >20 weeks. These well-defined criteria ensured accuracy and consistency in assessing and reporting the pregnancy outcomes throughout the study.

Statistical analysis

We used Stata 17 to analyze data. In terms of baseline features, including age, BMI (body mass index), and AMH (anti-Müllerian hormone) were recorded as mean and standard deviation (SD). We also reported the number of failed cycles and the number of failed embryos. The student t-test was used to compare continuous variables between groups, whereas the chi-squared test with Bonferroni correction was employed to compare categorical variables. A p-value of <0.05 was considered significant.

## Results

In this retrospective cohort study, we compared the reproductive outcomes of 69 RIF patients who had pET and 74 RIF patients who had sET. Detailed patient characteristics are described in Table 1. The data revealed no significant differences between the pET and sET groups regarding age, BMI, AMH levels, type of infertility, and the previous failed cycles.

**Table 1:** The patient characteristics of the control and experimental groups pET: personalized embryo transfer group sET: standard embryo transfer group SD: standard deviation A P value of <0.05 was considered significant

Patient characteristics	pET (n=69)	sET (n=74)	P value
Age, years (mean ± SD)	33.5 ± 5.2	32.0 ± 4.3	0.076
BMI (body mass index), kg/m^2^ (mean ± SD)	22.4 ± 2.8	21.9 ± 3.2	0.295
AMH (anti-Müllerian hormone), ng/mL (mean ± SD)	4.2 ± 3.3	4.1 ± 2.9	0.750
Type of infertility			0.213
1/ Primary infertility, n (%)	54 (78.3%)	50 (67.6%)	
2/ Secondary infertility, n (%)	15 (21.7%)	24 (32.4%)	
Previous failed embryo transfer cycles (mean ± SD)	3.6 ± 1.5	3.3 ± 1.0	0.437
Previous failed embryos (mean ± SD)	5.3 ± 3.2	4.4 ± 1.6	0.503

Table 2 presents the results of the Genratest conducted on a total of 69 patients undergoing fertility treatments. Generally, the Genratest can identify approximately 89.9% (n=62/69) of the displaced WOI in patients with RIF. Among the patients, approximately 10.1% (n=7/69) were classified as "receptive". The majority of patients fell under the "non-receptive" category, which further comprised two subgroups: "pre-receptive" (n=49/62, 79%) and "post-receptive" (n=13/62, 21%).

**Table 2:** Genratest results in identifying the displaced window of implantation (WOI) in patients with recurrent implantation failure (RIF) WOI: window of implantation RIF: recurrent implantation failure

Total (n = 69)	Frequency (n)	Percentage (%)
Receptive	7	7/69 (10.1%)
Non-receptive	62	62/69 (89.9%)
1/ Pre-receptive	49	49/62 (79%)
2/ Post-receptive	13	13/62 (21%)

Table 3 compares pregnancy outcomes in patients who underwent pET and sET. In the pET group, 36 out of 69 patients (52.2%) achieved clinical pregnancy, and in the sET group, 35 out of 74 patients (47.3%) achieved clinical pregnancy. The P value was 0.679, indicating no significant difference between the groups. Similarly, ongoing pregnancy rates showed no significant difference between pET (n=28/69, 40.6%) and sET (n=30/74, 40.5%), with a p-value of 0.996. Regarding live birth rates, the pET group had 26 out of 69 patients (37.7%) achieving a live birth, while the sET group had 22 out of 74 patients (29.7%) achieving a live birth. The P value was 0.407, indicating no significant difference between the groups. Moreover, the incidence of miscarriage was comparable between pET (n=9/69, 13%) and sET (n=11/74, 14.9%), with a P value of 0.942.

**Table 3:** The clinical outcomes of personalized embryo transfer (pET) and standard embryo transfer (sET) pET: personalized embryo transfer group sET: standard embryo transfer group SD: standard deviation A P value of < 0.05 was considered significant

Characteristics	pET (n=69)	sET (n=74)	P value
Endometrial thickness, mm (mean ± SD)	10.5 ± 1.4	9.6 ± 1.2	<0.001
The number of transferred embryos (mean ± SD)	1.5 ± 0.5	1.7 ± 0.4	0.001
The number of good embryos transferred (mean ± SD)	0.8 ± 0.6	0.9 ± 0.8	0.495
Embryo age			0.682
1/ Day 3, n (%)	11 (15.9%)	9 (12.2%)	
2/ Day 5, n (%)	58 (84.1%)	65 (87.8%)	
Clinical pregnancy, n (%)	36 (52.2%)	35 (47.3%)	0.679
Ongoing pregnancy, n (%)	28 (40.6%)	30 (40.5%)	0.996
Live birth, n (%)	26 (37.7%)	22 (29.7%)	0.407
Miscarriage, n (%)	9 (13%)	11 (14.9%)	0.942

For further subgroup analysis, we categorized the population into receptive (n=7) and non-receptive (n=62) WOI groups. Additionally, we also performed another subgroup analysis between pre-receptive (n=49) and post-receptive (n=13) WOI groups. However, no significant differences were observed in pregnancy outcomes between these subgroups. The detailed information is described in Table 4 and Table 5.

**Table 4:** The clinical outcomes of patients with receptive and non-receptive Genratest results WOI: window of implantation SD: standard deviation A P value of <0.05 was considered significant

Characteristics	Receptive WOI (n = 7)	Non-receptive WOI (n = 62)	P value
Endometrial thickness, mm (mean ± SD)	11.3 ± 1.4	10.4 ± 1.4	0.136
The number of transferred embryos (mean ± SD)	1.4 ± 0.5	1.5 ± 0.5	0.854
The number of good embryos transferred (mean ± SD)	1.0 ± 0.6	0.8 ± 0.6	0.352
Embryo age			<0.001
1/ Day 3, n (%)	1 (14.3%)	10 (16.1%)	
2/ Day 5, n (%)	6 (85.7%)	52 (83.9%)	
Clinical pregnancy, n (%)	2 (28.6%)	34 (54.8%)	0.358
Ongoing pregnancy, n (%)	2 (28.6%)	26 (41.9%)	0.782
Live birth, n (%)	1 (14.3)	25 (40.3)	0.349
Miscarriage, n (%)	0 (0)	9 (14.5)	0.625

**Table 5:** The clinical outcomes of patients with pre-receptive window of implantation (WOI) and post-receptive WOI WOI: window of implantation SD: standard deviation A P value of < 0.05 was considered significant

Characteristics	Pre-receptive WOI (n = 49)	Post-receptive WOI (n = 13)	P value
Endometrial thickness, mm (mean ± SD)	10.3 ± 1.4	10.7 ± 1.4	0.322
The number of transferred embryos (mean ± SD)	1.5 ± 0.5	1.4 ± 0.5	0.509
The number of good embryos transferred (mean ± SD)	0.8 ± 0.6	0.7 ± 0.6	0.621
Embryo age			0.176
1/ Day 3, n (%)	10 (20.4%)	0 (0%)	
2/ Day 5, n (%)	39 (79.6%)	13 (100%)	
Clinical pregnancy, n (%)	28 (57.1%)	6 (46.2%)	0.693
Ongoing pregnancy, n (%)	21 (42.9%)	5 (38.5%)	0.99
Live birth, n (%)	20 (40.8%)	5 (38.5%)	0.99
Miscarriage, n (%)	8 (16.3%)	1 (7.7%)	0.732

## Discussion

In this research, we present a comprehensive retrospective analysis of Ge thenratest in women with RIF. Based on patient characteristics, data indicate that both groups were well-matched in terms of clinical factors. However, pET did not lead to significant improvements in clinical pregnancy, ongoing pregnancy, live birth, or miscarriage rates compared to the sET approach. Although the live birth rate in the pET group (n=26/69, 37.7%) was higher than the sET group (n=22/74, 29.7%), the P value of 0.407 suggests that this difference is not statistically significant. This means that the observed difference in live birth rates between the two groups could have occurred by chance, and there is no strong evidence to support the widespread use of pET guided by Genratest. Our findings are also consistent with a previous study [13]. Conversely, some studies also support the utilization of the Genratest in women with RIF [6,7].

The limitations of the Genratest have been discussed in a related study [9]. To address these limitations in our population, we have highlighted some key evidence. First, endometrial receptivity can vary not only between individuals but also within the same individual across different menstrual cycles [14]. In our study, one patient experienced this intra-patient variability and had to repeat the Genratest three times, leading to increased financial costs for the patient. Second, the success of personalized embryo transfer based on Genratest results may depend on factors beyond the endometrial receptivity status [15,16]. For instance, the quality of the embryos transferred, the patient's age, BMI, and ovarian reserve can significantly impact pregnancy outcomes. Although we did not find significant differences in these factors between the pET and sET groups, there might be other unmeasured variables or unknown interactions that could be contributing to the lack of a significant difference in pregnancy rates. Finally, RIF patients are more willing to try Genratest as a new hope, especially when the source of viable embryos is limited. However, it is essential to recognize that the causes of RIF can vary widely, and displaced WOI may not be the main cause of unexplained RIF in our population. Regarding the subgroup analysis, the results between the receptive and non-receptive WOI groups, as well as between the pre-receptive and post-receptive WOI groups, did not reveal any significant differences in pregnancy outcomes. This further emphasizes the need to understand patient-specific factors that can influence the Genratest's success.

Our study also has some limitations. First, we acknowledge that the retrospective nature of our investigation, the lack of randomization, and the relatively small sample size may limit the statistical power to detect differences in pregnancy outcomes. This limitation has the potential to introduce bias and may impact the generalizability of our findings. Second, the study was conducted at a single center, which raises concerns about the external validity of the results. Lastly, the variation in the definition of RIF among IVF centers may lead to different strategies for patient recruitment. To establish more robust evidence on the utility of ERA-guided pET, prospective and randomized controlled trials involving multiple centers and larger sample sizes are needed. Additionally, achieving a worldwide consensus on the RIF definition is essential for ensuring consistent research outcomes. Moreover, further research also needs to explore the economic analysis of the Genratest.

In conclusion, while the Genratest shows promise in identifying the displaced WOI in RIF patients, our study's findings highlight the complexity of endometrial receptivity and its impact on pregnancy outcomes. Future research should focus on investigating the underlying mechanisms contributing to the Genratest's efficacy and identifying specific patient subgroups that may benefit from pET. Additionally, there is a need for further investigations into the potential role of other biomarkers or approaches in assessing endometrial receptivity. Multidisciplinary research is crucial to develop novel strategies that can enhance ART success rates for RIF women. These efforts will not only improve our understanding of endometrial receptivity and implantation mechanisms but also contribute to refining and advancing ART protocols.

## Conclusions

The pET approach based on the results of the Genratest did not show a significant improvement in pregnancy outcomes, including clinical pregnancy, ongoing pregnancy, live birth, or miscarriage rates. Further research is needed to identify the role of the Genratest in RIF patients.
