# Correction: Assessing the Impact of Genratest on Women With Recurrent Implantation Failure: A Single-Center Study

**DOI:** 10.7759/cureus.c156

**Published:** 2024-01-25

**Authors:** Huy Phuong Tran, Loc Thai Ly, Vy Nguyen-Thao Do, Tuyet Thi-Diem Hoang, Thuy Thi-Thanh Tran, Hien Nguyen-Trong Le, Phuong Thi-Vy Nguyen, Ngoc Anh Nguyen, Trang Nguyen-Khanh Huynh

**Affiliations:** 1 Infertility Department, Hung Vuong Hospital, Ho Chi Minh, VNM; 2 Medical Genetics Department, Hung Vuong Hospital, Ho Chi Minh, VNM; 3 Obstetrics and Gynaecology Department, Pham Ngoc Thach University of Medicine, Ho Chi Minh, VNM

This article has been corrected to address any potential confusion regarding the use of the term "endometrial receptivity array" and the "ERA" abbreviation. The intent of the authors was to use ERA as an abbreviation of endometrial receptivity array and they did not intend to imply any association with the ERA Endometrial Receptivity Analysis or Array tests by Igenomix. However, due to the trademark on this term owned by Igenomix, the authors have agreed to replace all instances of "endometrial receptivity array" and "ERA" with "Genratest". In addition, the Study Protocol section, second paragraph, second sentence has been revised to read as follows: "The samples were then shipped at room temperature to GENTIS Company for Genratest analysis to identify the WOI."

